# Epidemiological and Serological Analysis of a SARS-CoV-2 Outbreak in a Nursing Home: Impact of SARS-CoV-2 Vaccination and Enhanced Neutralizing Immunity Following Breakthrough Infection

**DOI:** 10.3390/microorganisms10091809

**Published:** 2022-09-09

**Authors:** Barbara I. Streibl, Heidi Lahne, Andreas Grahl, Philipp Agsten, Magdalena Bichler, Christa Büchl, Marco Damzog, Ute Eberle, Stefan Gärtner, Bernhard Hobmaier, Gabriele Margos, Martin Hoch, Sabrina Jungnick, Walter Jonas, Katharina Katz, Liane Laubert, Barbara Schutt, Cornelia Seidl, Bianca Treis, Daniel Weindl, Karen Zilch, Manfred Wildner, Bernhard Liebl, Nikolaus Ackermann, Andreas Sing, Volker Fingerle

**Affiliations:** 1 Public Health Microbiology Unit, Bavarian Health and Food Safety Authority, 85764 Oberschleißheim, Germany; 2 Health Office Neumarkt, 92318 Neumarkt, Germany

**Keywords:** SARS-CoV-2 Alpha variant, nursing home, outbreak, breakthrough infection, Comirnaty® COVID-19 vaccine, epidemiology, serology, SARS-CoV-2 antibodies, neutralizing antibodies

## Abstract

**Background**: Despite a vaccination rate of 82.0% (*n* = 123/150), a SARS-CoV-2 (Alpha) outbreak with 64.7% (*n* = 97/150) confirmed infections occurred in a nursing home in Bavaria, Germany. **Objective**: the aim of this retrospective cohort study was to examine the effects of the Corminaty vaccine in a real-life outbreak situation and to obtain insights into the antibody response to both vaccination and breakthrough infection. **Methods**: the antibody status of 106 fully vaccinated individuals (54/106 breakthrough infections) and epidemiological data on all 150 residents and facility staff were evaluated. **Results**: SARS-CoV-2 infections (positive RT-qPCR) were detected in 56.9% (*n* = 70/123) of fully vaccinated, compared to 100% (*n* = 27/27) of incompletely or non-vaccinated individuals. The proportion of hospitalized and deceased was 4.1% (*n* = 5/123) among fully vaccinated and therewith lower compared to 18.5% (*n* = 5/27) hospitalized and 11.1% (*n* = 3/27) deceased among incompletely or non-vaccinated. Ct values were significantly lower in incompletely or non-vaccinated (*p* = 0.02). Neutralizing antibodies were detected in 99.1% (*n* = 105/106) of serum samples with significantly higher values (*p* < 0.001) being measured post-breakthrough infection. α-N-antibodies were detected in 37.7% of PCR positive but not in PCR negative individuals. **Conclusion**: Altogether, our data indicate that SARS-CoV-2 vaccination does provide protection against infection, severe disease progression and death with regards to the Alpha variant. Nonetheless, it also shows that infection and transmission are possible despite full vaccination. It further indicates that breakthrough infections can significantly enhance α-S- and neutralizing antibody responses, indicating a possible benefit from booster vaccinations.

## 1. Introduction

In May 2021, a cluster of SARS-CoV-2 cases arose in a nursing home in Bavaria (Germany) despite a high vaccination rate of 82.0%. All cases were attributed to the Alpha variant (B.1.1.7), which as summarized by Rotondo et al. is more infectious, characterized by increased viral loads and more often leads to hospitalization compared to original strains [1].

Compared to the general population, residents of nursing homes and care facilities have an increased risk of severe disease progression and mortality. In addition to advanced age and existing comorbidities, the increased risk results from frequent close contact with other residents as well as nursing staff [2,3]. Residents also have a significantly increased risk of infection compared to the same age population who are not cared for in nursing homes [4,5,6]. According to a Canadian study, the risk of dying from COVID-19 before vaccination was increased 13.1-fold in nursing home residents over 69 years of age compared to people of the same age not living in such facilities [7]. According to studies on COVID-19 measures in long-term care in both Europe and Germany based on data from 2020, about half of all SARS-CoV-2-related deaths were residents in nursing homes [8,9].

There is also an increased risk of SARS-CoV-2 infection among nursing home staff. A study that investigated SARS-CoV-2 seropositivity in health care personnel found that seroprevalence among nursing home staff was significantly higher than among hospital staff [10]. Furthermore, there is the additional risk of an undetected infection among staff members that maybe unknowingly transmitted to residents. Even with the infection control and contact restriction measures that might be in place, they cannot always be fully adhered to, as close physical contact is often required in the context of nursing activities [8].

All of this was taken into account in the German vaccination prioritisation, according to which first priority was given to persons over 80 years of age, residents of nursing homes and employees in direct contact with residents [11]. In the investigated nursing home, residents and staff were offered the vaccination in January 2021, which was accepted by almost 88% of residents and 59% of employees. At the time of the outbreak, first reports on SARS-CoV-2 vaccination breakthrough infections were published [12,13]. However, it was still unclear to what extent vaccination may prevent virus transmission. Thus, the high number of SARS-CoV-2 infections recorded in the nursing home despite a vaccination rate of 82% promped an investigation within the framework of the German Infection Protection Act.

The aim of this retrospective cohort study was to examine the risks of infection, hospitalization and death due to SARS-CoV-2 in fully vaccinated compared to incompletely or non-vaccinated individuals in a real world nursing home setting. It also included the determination of antibody status with a focus on neutralizing antibodies as determined by surrogate neutralization assay to obtain insights into the humoral immune response of nursing home residents and staff to both vaccination and breakthrough infection. A preliminary analysis, which focused on the epidemiological impact of the Comirnaty vaccination, has been published in German in a German public health letter [14], due to its relevance for future containment strategies. The results reported here represent a more comprehensive analysis with a focus on the antibody response.

## 2. Materials and Methods

### 2.1. Sample Collection and Composition of the Cohort

In the period between 2 May 2021 and 6 June 2021, officials from the responsible health office tested all of the 150 people affiliated with the affected nursing home, including 121 residents and 29 staff members, by real-time quantitative polymerase chain reaction (RT-qPCR) as part of the outbreak containment strategy. RT-qPCR was performed in an external laboratory using the COBAS 6800 (target gene: ORF). Cycle threshold values (Ct values) were used as correlate for viral load.

In total, 82% (*n* = 123) of the 150 individuals had been fully vaccinated with the Comirnaty vaccine (Pfizer–BioNTech). Vaccinations were carried out for all individuals on the following dates: first vaccination: 11/16 January 2021, second vaccination: 6 Feburary 2021. All SARS-CoV-2 infections determined by positive RT-PCR that occurred at least 14 days after full vaccination were considered breakthrough infections. Data of the local health office was reviewed to determine whether a prior SARS-CoV-2 infection had been recorded for any of the 150 individuals.

Following informed consent, 7.5 mL blood samples were taken from 106 fully vaccinated individuals on site by mobile teams of the Bavarian Health and Food Safety Authority (LGL) on 7 June 2021, immediately transported to the LGL and stored at 7 °C until testing. Additional data was collected for all 150 individuals involved in the outbreak, such as infection and vaccination status, age, sex, Ct values and the need for hospitalization or death resulting from a SARS-CoV-2 infection. All 150 individuals were included in the epidemiological analysis. Inclusion into the serological analysis was subject to the patient’s consent to give blood.

### 2.2. Determination of the Antibody Status

All tests were performed and evaluated according to the manufacturer’s instructions. All tests are summarized in Table 1.

### 2.3. Statistical Data Analysis

SPSS version 25 was used for statistical analysis of epidemiological data. Python 3.8.8 was used for data visualisation and statistical analysis of serological data. Unvaccinated and incompletely vaccinated individuals were combined into one group “incompletely or non-vaccinated”. Relative risks (RR) with 95% confidence intervals were calculated to compare risks for specific parameters such as infection, hospitalization or death between fully vaccinated and incompletely or non-vaccinated individuals. Since the measured antibody and Ct values did not follow a normal distribution, the parameter-free Wilcoxon rank sum test was used to determine statistical differences. Accordingly, the Spearman rank correlation coefficient was used to investigate correlations. A significance level of *p* < 0.05 was assumed in all calculations.

## 3. Results

### 3.1. Vaccination Status and Infection

**Figure 1:**
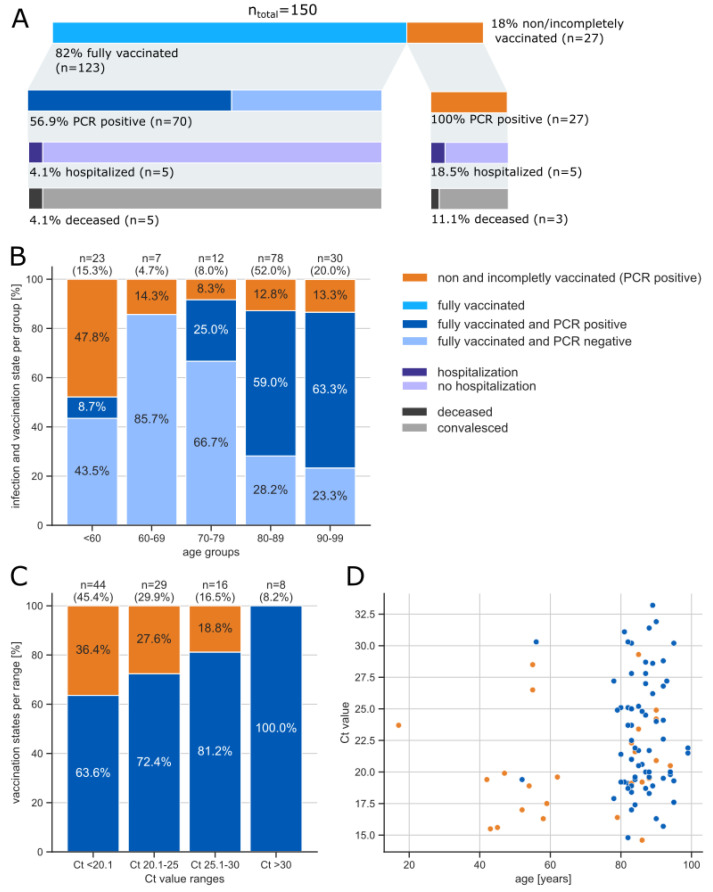
(**A**) Overview of the individuals considered in the investigated outbreak outlining the share of fully vaccinated and incompletely or non-vaccinated and the proportion of hospitalized and deceased by vaccination status. (**B**) Vaccination and infection status by age group. Fully vaccinated individuals can be divided into PCR positive (dark blue) and PCR negative (light blue) while all incompletely or non-vaccinated were PCR positive (orange). (**C**) Proportion of incompletely or non-vaccinated (orange) among PCR positive individuals in each Ct value group (fully vaccinated in blue). (**D**) Ct value distribution in relation to age among fully vaccinated (*n* = 123, blue) and incompletely or non-vaccinated (*n* = 27, orange).

Vaccination and infection status as determined by SARS-CoV-2 RT-qPCR were recorded for all 150 individuals (121 residents and 29 staff members) involved in the investigated outbreak (Figure 1A). The investigated cohort consisted of 96.0% (*n* = 144) women and 4.0% (*n* = 6) men. The median age was 84 years (range: 17–99 years, IQR: 78–88.75 years), with the age group 80–89 representing the largest proportion with 52.0% (*n* = 78) (Figure 1B).

Of the 150 individuals, 82.0% (*n* = 123) were fully vaccinated, 0.7% (*n* = 1) vaccinated once and 17.3% (*n* = 26) were non vaccinated. The percentage of fully vaccinated individuals was highest among the 70–79 year olds with 91.7%, and with 52.2% (*n* = 12/23), by far the lowest, among the age group under 60. This age group was composed of 95.7% (*n* = 22/23) employees. Overall, the vaccination coverage among staff members (*n* = 29) was 58.6%. None of the individuals had been diagnosed with a SARS-CoV-2 infection prior to May 2021.

In total, 64.7% (*n* = 97/150) of residents and staff members tested positive for SARS-CoV-2 in the period between May and June 2021. Among the incompletely or non-vaccinated, 100% (*n* = 27/27) tested positive in PCR, while only 56.9% (*n* = 70/123) were PCR positive among the fully vaccinated (Figure 1A). This results in a relative risk of RR = 1.76 (95% CI: 1.51–2.05) for the incompletely or non-vaccinated to acquire a SARS-CoV-2 infection compared to the fully vaccinated in the investigated setting. It is noteworthy that the proportion of infected individuals among the fully vaccinated increased with age: from 27.3% in the age group 70–79 years to 73.1% in the highest age group 90–99 (Figure 1B). Two individuals under 60 were PCR-positive despite vaccination (16.7% of fully vaccinated), one individual being a resident and one being staff.

The majority of SARS-CoV-2-infected individuals (89.7%, *n* = 87/97), exhibited a mild or even asymptomatic course of infection that did not require hospitalization. Of 150 residents and staff members, 6.7% (*n* = 10) had to be hospitalized due to SARS-CoV-2 infection. These were 4.3% (*n* = 1/23) in the age group <60 years, 9.0% of those aged 80–89 years (*n* = 7/78), 6.7% (*n* = 2/30) of those aged 90–99 years). The proportion of hospitalized individuals among the fully vaccinated was at 4.1% (*n* = 5/123) significantly lower compared to the incompletely or non-vaccinated at 18.5% (*n* = 5/27) (Figure 1A). This results in a relative risk of RR = 4.6 (95% CI: 1.4–14.6) for the incompletely or non-vaccinated to be hospitalized due to a SARS-CoV-2 infection compared to the fully vaccinated in the investigated setting. When considering only residents but not staff, the proportion of hospitalized amounted to 4.6% (*n* = 5/108) among fully vaccinated residents compared to 26.7% (*n* = 4/15) among incompletely or non-vaccinated residents.

In total, 5.3% (*n* = 8/150) of residents, all female aged 83 to 99 years, had died. Among them, 62.5% (*n* = 5) were fully vaccinated and 37.5% (*n* = 3) were incompletely or non-vaccinated. Only 5 of the 8 deceased residents had been hospitalized (3 fully vaccinated and 2 incompletely or non-vaccinated), and the other 3 died in the nursing home (2 fully vaccinated and 1 incompletely or non-vaccinated). The proportion of deceased among the fully vaccinated was lower at 4.1% (*n* = 5/123) than the proportion of deceased among the incompletely or non-vaccinated at 11.1% (*n* = 3/27) (Figure 1A). Thus, we found a relative risk of RR = 2.7 (95% CI: 0.7–10.8) for death from SARS-CoV-2 infection among the incompletely or non-vaccinated compared to the fully vaccinated. When considering only residents, the proportion of deceased amounted to 4.6% (*n* = 5/108) among fully vaccinated residents compared to 20% (*n* = 3/15) among incompletely or non-vaccinated residents.

The Ct values of all 97 PCR-positive individuals ranged from Ct14.6 to Ct33.2 with a median Ct value of Ct21.0 (IQR: Ct19.1–Ct25.0). A total of 45.4% (*n* = 44/97) had very low Ct values, ≤Ct20, indicating very high viral loads. Only 8.2% (*n* = 8/97) exhibited Ct values above Ct30. Considering Ct values with regards to vaccination status, it becomes apparent that the proportion of individuals with a high viral load is significantly (Wilcoxon rank sum test: *p* = 0.02, effect size r = 0.2) lower among the fully vaccinated compared to the incompletely or non-vaccinated. The largest proportion of incompletely or non-vaccinated was found in the category with the highest viral loads with Ct values ≤ 20 (36.4%, *n* = 16/44), while the category with Ct values > 30 was composed entirely from fully vaccinated individuals (*n* = 8) (Figure 1C). A slight though non-significant correlation (r = 0.15, *p* = 0.14) was observed between Ct values and age, with infected individuals younger than 60 tending to have higher viral loads (Figure 1D). This observation can most likely be attributed to a clustering effect, with many of those under 60 years not having been vaccinated, while the majority of individuals over 60 years had been fully vaccinated.

### 3.2. Serological Results

**Figure 2:**
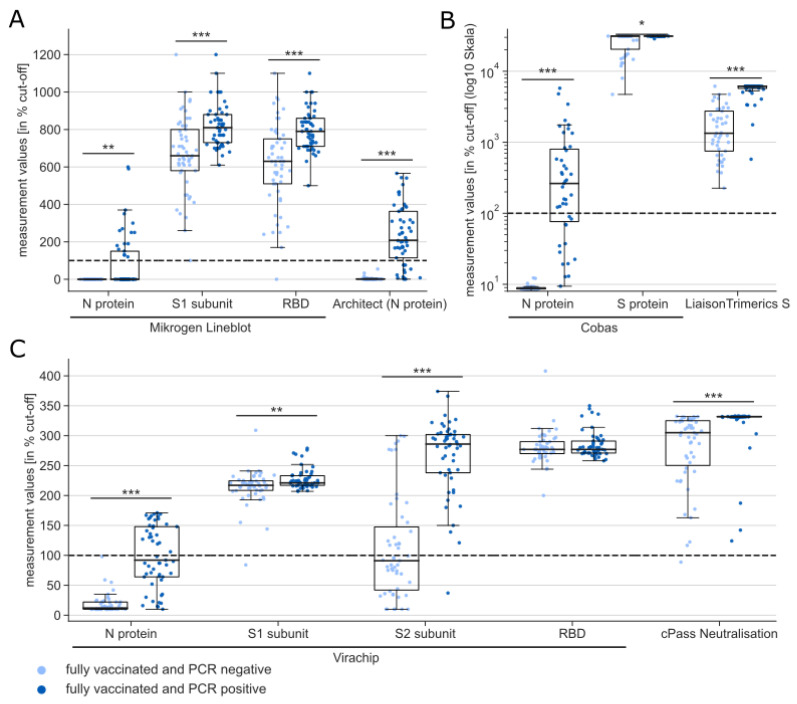
Measurements of all antibody assays performed represented as percent of the cut-off value (indicated by dashed line), subdivided by infection status (PCR positive: dark blue, PCR negative light blue). Wilcoxon rank sum test with *: *p* < 0.05, **: *p* < 0.01, ***: *p* < 0.001. (**A**) Mikrogen Lineblot with antigens: N protein, S1 subunit and RBD, Architect with N protein as antigen, (**B**) Cobas N Protein, Cobas S Protein and Liaison TrimericS S Protein, plotted on a logarithmic scale for better representation of the wide range of measured values, and (**C**) Virachip with N protein, S1 subunit, S2 subunit and RBD as antigens and cPass surrgat neutralization assay.

Serum samples for serological investigations were obtained from 106 of the 123 fully vaccinated persons, 50.0% (*n* = 53) of which were PCR-positive. Direct SARS-CoV-2 antibody detection was performed using seven different tests (Figure 2).

All examined individuals had produced antibodies against the Spike glycoprotein (hereafter referred to as α-S-antibodies) and the receptor binding domain (α-RBD-antibodies) (Figure 2A: Mikrogen Lineblot α-S1 subunit and α-RBD, Figure 2B: Cobas α-S protein and LiaisonTrimericS α-S protein, Figure 2C: Virachip α-S1 subunit, α-S2 subunit and α-RBD). However, the reaction against the S2 subunit appeared to be weaker compared to the S1 subunit and the RBD. α-S-antibodies can be produced as a response towards both vaccination and natural infection. Antibodies against the nucleocapsid protein (α-N-antibodies), which is not part of the vaccination, were not detectable in any of the PCR-negative tested vaccinated individuals (Figure 2A: Mikrogen Lineblot N protein and Architect, Figure 2B: Cobas and Figure 2C: Virachip N protein). The fraction of PCR-positive vaccinated individuals for whom α-N-antibodies were detected ranged between 16.0% (Mikrogen Lineblot N protein) and 37.7% (Architect). In 99.1% (*n* = 105/106) of examined individuals, neutralizing antibodies had been detected. Both neutralizing antibodies and α-S-antibodies were significantly higher in individuals who underwent natural infection following vaccination compared to PCR-negative individuals in all assays except for the Virachip α-RBD.

**Figure 3:**
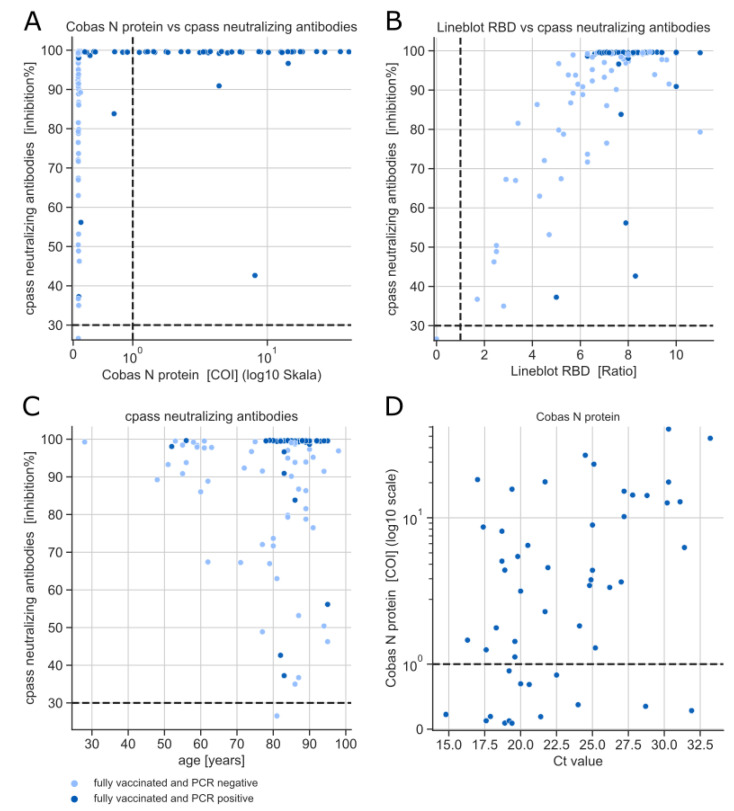
(**A**) Correlation of neutralizing antibodies with α-N antibodies (exemplified by Cobas N protein), Spearman Rank correlation coefficient r = 0.648 (*p* < 0.001) (all samples), r = −0.025 (*p* = 0.859) (PCR Negatives) and r = 0.277 (*p* = 0.045) (PCR Positives), (**B**) correlation of neutralizing antibodies with α-RBD antibodies (exemplified by Mikrogen Lineblot), Spearman Rank correlation coefficient r = 0.587 (*p* < 0.001) (all samples), r = 0.713 (*p* < 0.001) (PCR Negatives) and r = 0.194 (*p* = 0.165) (PCR Positives), (**C**) correlation of neutralizing antibodies with age. Spearman rank correlation coefficient r = 0.062 (*p* = 0.531) (all samples), r = −0.321 (*p* = 0.019) (PCR Negatives) and r = −0.141 (*p* = 0.323) (PCR Positives). (**D**) correlation of α-N antibodies (exemplified by Cobas N protein) with Ct values, Spearman Rank correlation coefficient r = 0.417 (*p* = 0.002) (PCR Positives only).

**Figure A1:**
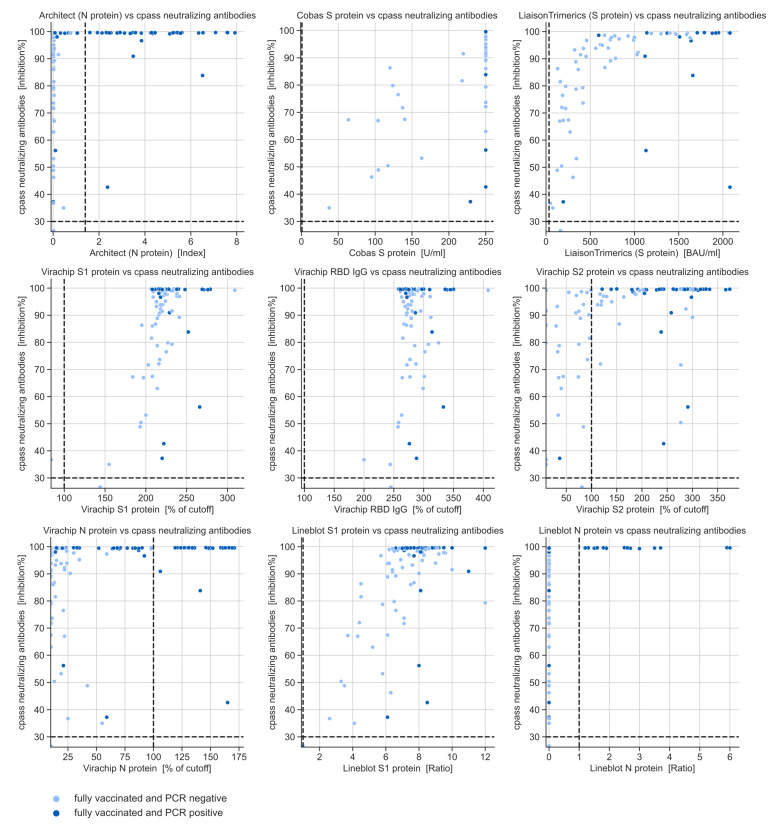
Relationship between measurement values for each assay with cPass neutralizing antibodies. Dashed line represents cut-off value. Corresponding Spearman Rank correlation coefficients are summarized in Table A1.

Due to a lack of knowledge on the extent to which protection against SARS-CoV-2 infection can be inferred from classical antibody detection, the correlations between the cPass surrogate neutralization assay and all other assays were investigated (Figure 3A,B and Figure A1). Almost all tests correlated moderately to strongly with the cPass, with a Spearmann correlation coefficient between r = 0.35 (Virachip S1) and r = 0.86 (Liaison TrimericS) for α-S-antibody assays and between r = 0.46 (Lineblot) and r = 0.67 (Cobas N protein) for α-N-antibody assays (compare Table A1). Despite this moderate correlation, no linear relationship can be observed between α-N-antibody assays and the cPass surrogate neutralization assay (Figure 3A and Figure A1). Instead, PCR-negative and PCR-positive samples form distinct clusters, with all PCR-negative individuals testing negative in all α-N-antibody assays and PCR-positive individuals presenting high neutralizing antibodies close to the detection limit of 100 inhibition%. Consistently, neutralizing antibodies and α-N-antibody assays did not correlate when examining PCR positives or PCR negatives individually. A moderate but statistically significant correlation (r = 0.58, *p* < 0.001) was evident between α-RBD antibodies (Mikrogen Lineblot) and neutralizing antibodies (Figure 3B). This correlation is stronger (r = 0.71, *p* < 0.001) when only PCR-negative individuals are considered. This holds true for all α-S or α-RBD antibody assays, with the exception of Virachip α-S2.

**Figure A2:**
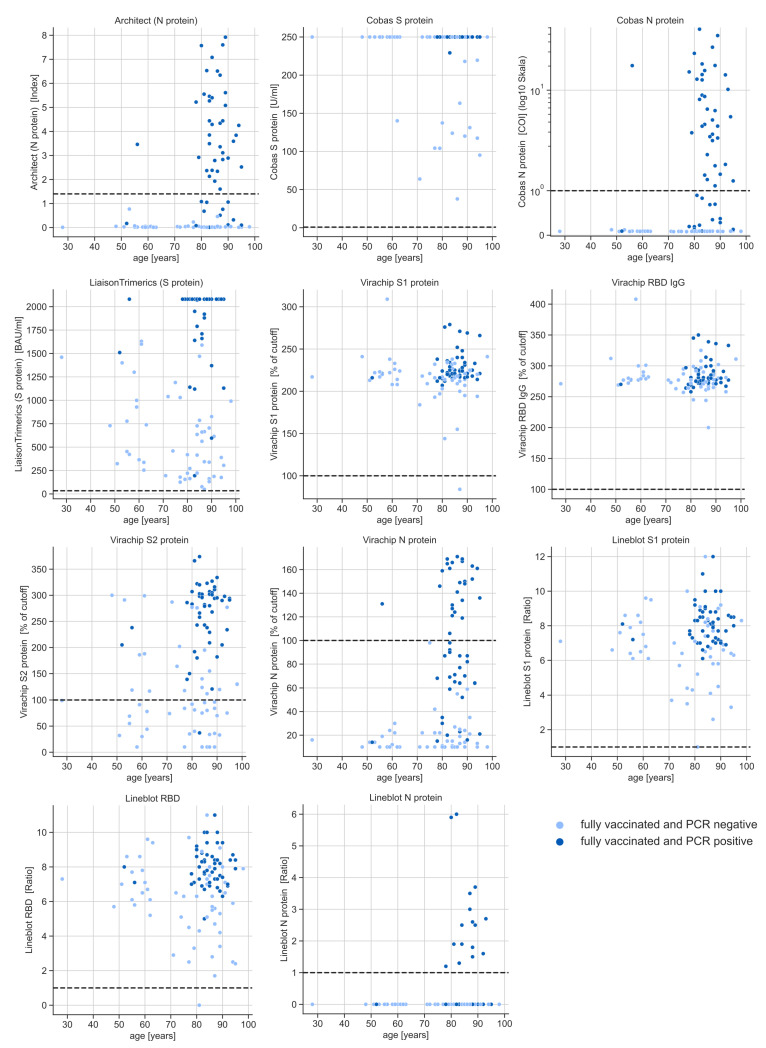
Relationship between measurement values for each assay with age. Dashed line represents cut-off value. Corresponding Spearman Rank correlation coefficients are summarized in Table A2.

Since it is recognized that the immune response becomes weaker with increasing age [15], the correlation between neutralizing antibody readings and age was examined (Figure 3C). In PCR-negative individuals, a low-to-moderate negative correlation (r = −0.32, *p* = 0.019) was observed. Again, due to high neutralizing antibodies levels in PCR-positive individuals, no correlation between age and neutralizing antibodies was observed in this subgroup (r = −0.14, *p* = 0.323) or when all samples were considered (r = 0.06, *p* = 0.531). Similar results were observed for α-S and α-RBD antibody assays (Figure A2 and Table A2). No correlation was found between α-N-antibodies and age (Figure A2 and Table A2).

**Figure A3:**
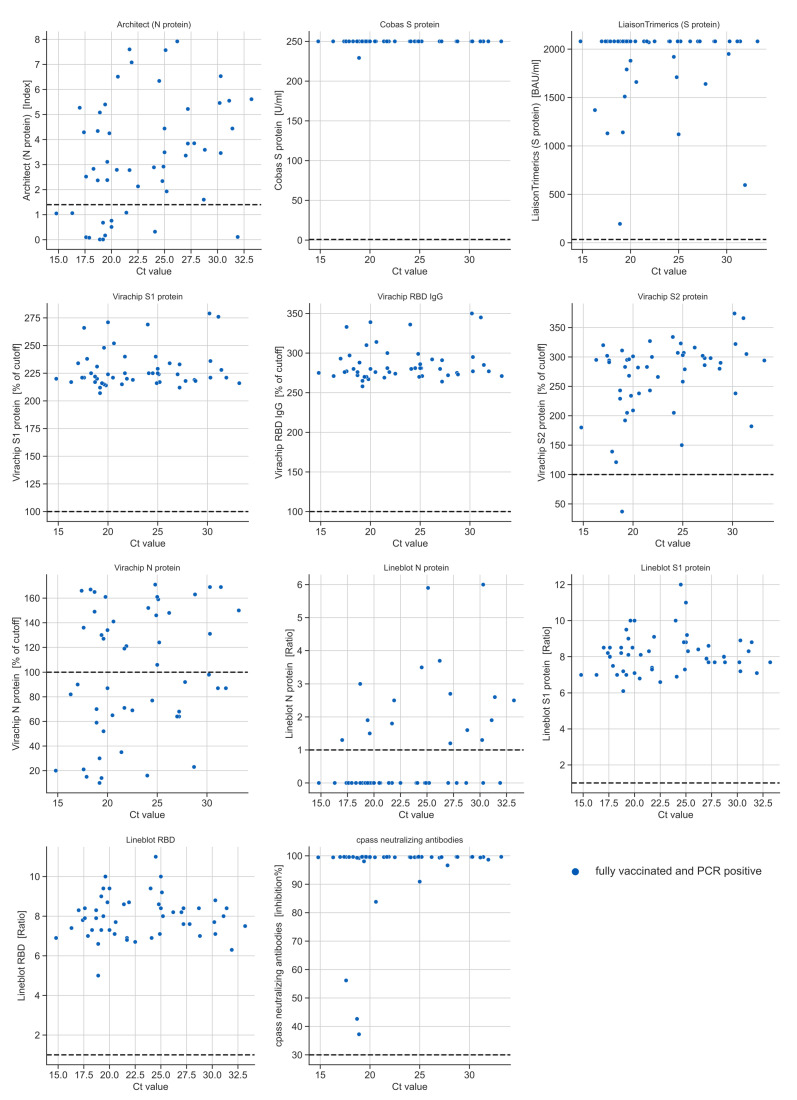
Relationship between measurement values for each assay with Ct values (PCR positive only). Dashed line represents cut-off value. Corresponding Spearman Rank correlation coefficients are summarized in Table A3.

**Figure A4:**
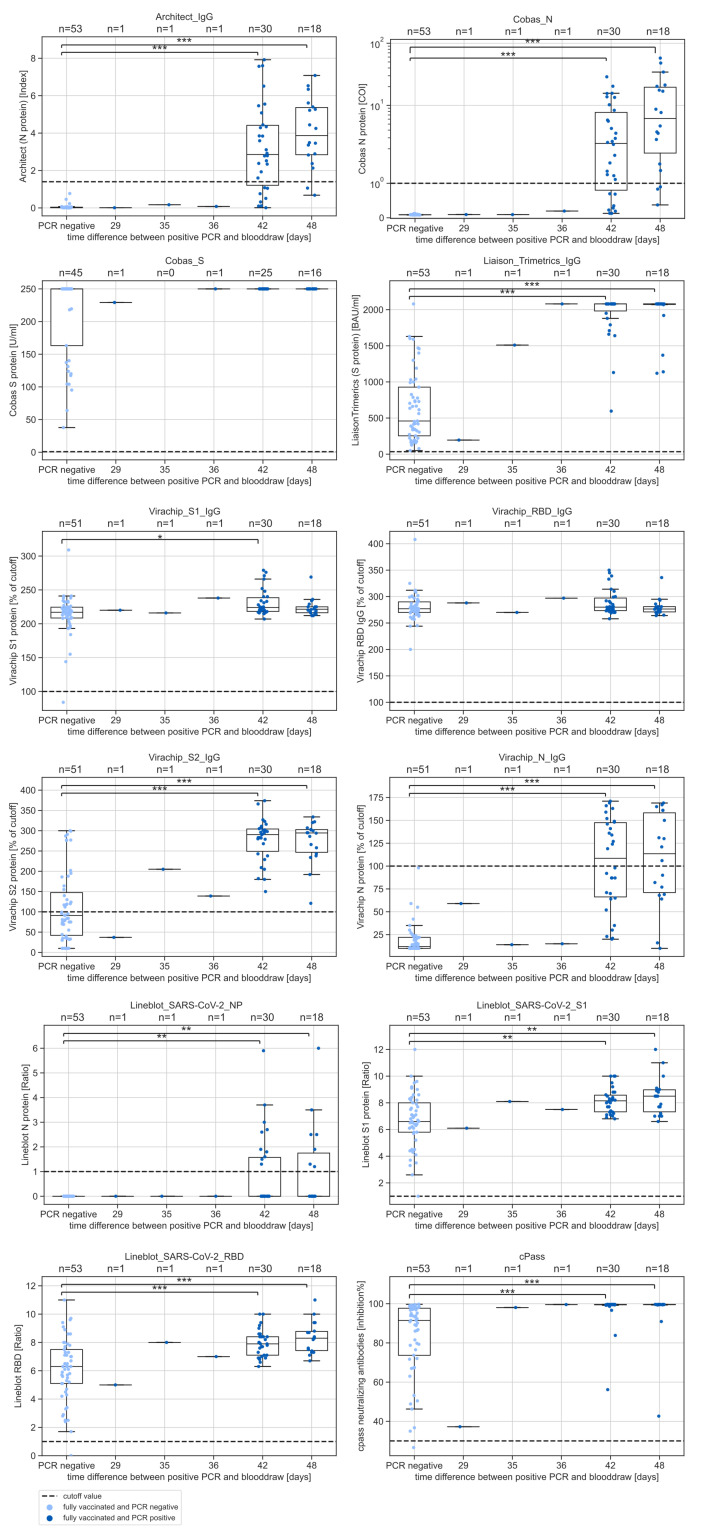
Measurement values for each assay in dependence of time difference between PCR and blood-draw. Dashed line represents cut-off value. Wilcoxon rank sum test with *: *p* < 0.05, **: *p* < 0.01, ***: *p* < 0.001.

Moderate correlations (e.g., Cobas N: r = 0.42, *p* = 0.002) were observed between Ct values and α-N-antibody assays (Figure 3D and Figure A3 and Table A3). However, three serum samples from individuals with high viral loads that tested negative for α-N-antibody were taken earlier relative to their positive PCR results (29, 35 and 36 days after PCR) than the rest of samples (day 42 and 48). Overall, no significant differences in antibody measurements were observed between samples taken on different days (Figure A4).

## 4. Discussion

### 4.1. Comirnaty Vaccination Protects against SARS-CoV-2 Alpha Variant in a Real Life Nursing Home Setting

This study reports real-life data from a SARS-CoV-2 Alpha variant outbreak in a nursing home in Bavaria, Germany, in May 2021. Despite a high proportion of SARS-CoV-2 infections observed even in fully vaccinated individuals, in the investigated setting the risk of infection, the viral load among infected, the risk of hospitalization and the risk of death had all been reduced by full Comirnaty vaccination. A differential selection bias of the vaccination status according to age groups can be assumed. Since, mainly, individuals in the age group <60 years were not vaccinated and working employees predominated in this age group (healthy worker effect), the risks for hospitalization and death related to a hypothetical population of nursing home residents of the same age are possibly higher than indicated here. Due to a lack of knowledge concerning individual risk situations (e.g., room occupancy, contact possibilities, pre-existing diseases), no other factors besides vaccination status were taken into account. Hence, the calculated risks should only be considered with the reservation of the influence of further potential risk factors. It should also be taken into account that the sample investigated only consisted of 150 individuals (121 residents and 29 staff). In addition to systematic selection effects of the occupancy of a single nursing home, random influences on the resident population and staff must be considered, which is why the results cannot be easily transferred to other nursing homes or even to the general population.

Nonetheless, in their basic statement the reported results are consistent with other published studies. An investigation on the effects of the Comirnaty vaccine in nursing homes in the Florence district (Italy) not only observed a severe drop in infection rates during the post-vaccination period but also significantly lower hospitalization and mortality rates [16]. Studies that investigated SARS-CoV-2 Alpha outbreaks in several French nursing homes post-Comirnaty vaccination reported reduced incidences (22.7 % vs. 43.7%) [17] and case-related mortalities (4.3% vs. 25% [17] and 6.5% vs. 25% [18]) among fully vaccinated residents compared to non-vaccinated residents. In a nationwide cohort of residents of long-term care facilities in Israel, vaccine effectiveness was 81.2% for SARS-CoV-2 infection and 85.3% for COVID-related death for full vaccination [19]. Additionally, an observational study in Bavaria (Germany) based on data obtained shortly before the investigated outbreak found a vaccine effectiveness of 68.3% for preventing SARS-CoV-2 infection, 73.2% for hospitalization, and 80.1% for mortality in the elderly (≥80 years) [20]. Rotondo et al. concluded in a review analysis on the efficacy of current vaccines that the efficacy against the Alpha variant is similar or only slightly reduced compared to the original Wuhan strain [1].

### 4.2. Breakthrough Infection Capable of Enhancing Vaccination-Induced Immune Response in Nursing Home Residents

The serological results indicate a significantly stronger α-S and neutralizing immune response in those who underwent SARS-CoV-2 infection post-vaccination. In individuals who had not undergone infection, the measured values for neutralizing antibodies correlated moderately negative with age, with strong variations being observed for individuals over 70 years of age, while for individuals of similar age with a verified infection values close to 100% inhibition were measured almost exclusively. Depending on their respective detection limits, the data from α-S-antibody assays (total α-S, α-S1, α-S2 and α-RBD) mostly followed similar patterns and correlated with the surrogate neutralization assay accordingly. These correlations are stronger when only PCR negative individuals are considered, which can likely be explained by the overall high neutralizing antibodies measured for PCR positive individuals.

So far, numerous studies have found that SARS-CoV-2 infection prior to vaccination is associated with a stronger immune response towards vaccination in nursing home residents [21,22,23,24,25,26,27] and also results in a slower decline in antibodies over time [21,22,28]. It has also been suggested that preceding infection was protective against breakthrough infections during Delta outbreaks in nursing homes [29,30]. Overall, our data suggest that breakthrough infections following vaccination are also capable of inducing an enhanced immune response, which has, to our knowledge, only been reported in one study so far, based on 10 infected nursing home residents of which 5 had been vaccinated [31].

No serological investigations exist from before this outbreak, but other investigations where serology was performed at outbreak onset found lower levels of α-S antibodies among infected compared to non-infected residents [32,33]. It is thus likely that the α-S immune response in PCR-positive individuals prior to breakthrough infection was as variable and possibly overall lower than in PCR-negative individuals. Taken together, this suggests that nursing home residents, despite age and waning immunity, will likely benefit from further booster vaccinations. Indeed, studies found that booster vaccination did increase antibody responses in nursing home residents both with and without prior COVID-19 infection [28]. Moreover, it particular increased Omicron specific neutralization [34]. Another study found superior infection-neutralizing capacity against all SARS-CoV-3 variants, including Omicron after three exposures, resulting from either three vaccinations or two vaccinations plus infection (post or prior to vaccination) [35]. These results are all in line with a reported vaccine effectiveness for the booster vaccination among nursing home residents of around 50% against infection [36,37] and 97.3% against SARS-CoV-associated death [37].

### 4.3. Low Infection-Specific Antibody Responses in Breakthrough Infections Possibly Correlate with Higher Viral Loads

No α-N-antibodies were detected in any of the PCR-negative vaccinated individuals, an indication that the outbreak had been well followed-up by PCR testing. However, since not all PCR-positive individuals produced α-N antibodies either, possible contact without the induction of an α-N response cannot be ruled out. It is also conceivable that an infection occurred too closely to the blood draw to elicit an α-N immune response. This might have been the case in the three earliest serum samples (days 29, 35 and 36 after PCR) in which no α-N-antibodies had been detected, while the majority of samples taken at least 40 days after PCR testing were positive for α-N-antibodies. Since blood samples were taken at one single time point, it is not possible to determine whether α-N-antibody-negative individuals might have subsequently produced α-N-antibodies. Nonetheless, this observation is in line with observations from the Spikevax (BioNTech/Pfizer) trials, where only 40% of infected individuals in the vaccine group had produced α-N-antibodies during the observation period compared to 93% in the placebo group [38], and observations from an Alpha outbreak in an Italian nursing home, where only 50% of residents had developed α-N-IgG 21 days later [39].

Moderate positive correlations were observed between Ct values and α-N-antibodies, which may suggest that individuals for whom higher α-N-antibody values were measured later might have had lower viral loads during the infection. This represents an observation contrary to Follman et al., who found higher viral loads in α-N seropositive individuals. While the three individuals providing the earliest serum samples all had Ct-values below 20, the overall correlation is likely not related to the time difference between infection and blood draw, since most samples taken between 42 and 48 days after diagnosis still exhibited a wide range of α-N-antibody values. More likely viral loads during infection and α-N seroconversion are both influenced by similar confounding factors such as immunosenescence or general immune deficiencies. It also has to be considered that the immune response is highly individual, with some individuals developing a strong humoral response and others relying more on cellular immunity.

### 4.4. Effect of Comirnaty Vaccination on Viral Loads Remains Uncertain

Since high viral loads were also detectable among vaccinated individuals, it can be assumed that vaccinated persons can be infectious. Indeed, several studies found no significant differences in viral loads among vaccinated and non-vaccinated health care workers [40,41,42]. Other studies, however, did find lower viral loads in recently fully vaccinated individuals during a Delta outbreak [43], significantly reduced infectious viral loads during Delta breakthrough infection in fully vaccinated individuals compared to unvaccinated individuals, and reduced infectious viral loads for Omicron breakthrough infection in boostered individuals compared to unvaccinated individuals [44]. This supports our finding of a significantly lower proportion of individuals with high viral loads among fully vaccinated compared to non-vaccinated individuals.

In the examined nursing home, the vaccination rate was considerably lower among staff members with 58.6% than among residents with 87.6%. Given the high viral loads (<Ct20) observed for the majority of unvaccinated younger individuals, vaccination for this group seems especially important in order to reduce the risk of transmission of SARS-CoV-2 to residents.

## 5. Conclusions

The presented results indicate that the Comirnaty vaccine did provide reliable protection against severe disease progression, but only limited protection against SARS-CoV-2 Alpha infection. Serological data indicate that breakthrough infections are able to induce an enhanced α-S and neutralizing immune response in nursing home residents and staff. Still, in order to protect the vulnerable group of nursing home residents, general protective measures such as keeping distance, observing hygiene rules, wearing masks, regular ventilation and repetitive testing seem advisable even after full vaccination of residents, staff and visitors. This applies even more in light of new variants with increased risk of transmission, such as the Omicron variant that has been prevalent in Germany since early 2022 [45]. 

## Figures and Tables

**Table 1:** Summary of all test kits used to determine α-SARS-CoV-2 antibody levels.

Test Kit	Manufacturer	Type of Test
Architect SARS-CoV-2 IgG Reagent Kit	Abbot, Sligo, Ireland	Chemiluminescence-based microparticle immunoassay (CMIA) for qualitative detection of IgG against the SARS-CoV-2 nucleocapsid (N)
Liaison SARS-CoV-2 TrimericS IgG	DiaSorin, Stillwater, USA	Chemiluminescence immunoassay (CLIA) for the quantitative detection of IgG against the spike glycoprotein (S)
Cobas Elecsys anti-SARS-CoV-2	Roche Diagnostics, Mannheim, Germany	Electrochemiluminescence-based immunoassay (ECLIA), detects antibodies directed against N regardless of antibody class
Cobas Elecsys anti-SARS-CoV-2 S	Roche Diagnostics, Mannheim, Germany	ECLIA, detects antibodies directed against S regardless of antibody class
SARS-CoV-2 ViraChip® IgG	Viramed Biotech AG, Planegg, Germany	Detection of IgG antibodies against SARS-CoV-2 S subunit 1 (S1), S2, receptor binding domain (RBD) and N (and against N of the four seasonal human coronaviruses 229E, NL63, OC43 and HKU1) in microarray format
Mikrogen recomLine SARS-CoV-2 IgG immunoassay	Mikrogen GmbH, Neuried, Germany	Lineblot for the detection of IgG antibodies against SARS-CoV-2 S1, RBD and N, (and against N of the four seasonal human coronaviruses)
cPass SARS-CoV-2 Neutralization Antibody Detection Kit	GeneScript, Nanjing City, China	Surrogate neutralization assay to detect potentially neutralizing antibodies against SARS-CoV-2 regardless of antibody class

## Data Availability

The data presented in this study are available on reasonable request from the corresponding authors. The data are not publicly available due to privacy restrictions.

## Abbreviations

The following abbreviations are used in this manuscript:

α-N-antibodies	antibodies directed against the nucleocapsid protein
α-RBD-antibodies	antibodies directed against the receptor binding domain
α-S-antibodies	antibodies directed against the spike glycoprotein
CI	confidence intervals
CLIA	chemiluminescence immunoassay
CMIA	chemiluminescence-based microparticle immunoassay
Ct values	cycle threshold values
ECLIA	electrochemiluminescence-based immunoassay
LGL	Bavarian Health and Food Safety Authority
N	nucleocapsid protein
PCR	polymerase chain reaction
RBD	receptor binding domain
RR	relative risk
RT-qPCR	real-time quantitative polymerase chain reaction
S	spike glycoprotein
S1	SARS-CoV-2 S subunit 1
S2	SARS-CoV-2 S subunit 2

## Appendix A

**Table A1:** Spearman correlation coefficients between all individual assays and cPass neutralizing antibody levels.

Assay	All Samples	PCR Positive	PCR Negative
Architect IgG	0.627 (*p* < 0.001)	0.250 (*p* = 0.071)	0.117 (*p* = 0.403)
Liaison Trimetrics IgG	0.857 (*p* < 0.001)	0.443 (*p* < 0.001)	0.884 (*p* < 0.001)
Cobas N-Protein	0.648 (*p* < 0.001)	0.277 (*p* = 0.045)	−0.025 (*p* = 0.859)
Cobas S-Protein	0.590 (*p* < 0.001)	0.261 (*p* = 0.091)	0.720 (*p* < 0.001)
Lineblot SARS-CoV-2 NP	0.426 (*p* < 0.001)	0.219 (*p* = 0.114)	
Lineblot SARS-CoV-2 RBD	0.587 (*p* < 0.001)	0.194 (*p* = 0.165)	0.713 (*p* < 0.001)
Lineblot SARS-CoV-2 S1	0.541 (*p* < 0.001)	0.220 (*p* = 0.113)	0.636 (*p* < 0.001)
Virachip S1 IgG	0.312 (*p* = 0.001)	−0.252 (*p* = 0.069)	0.689 (*p* < 0.001)
Virachip RBD IgG	0.043 (*p* = 0.661)	−0.260 (*p* = 0.060)	0.315 (*p* = 0.024)
Virachip S2 IgG	0.670 (*p* < 0.001)	0.221 (*p* = 0.112)	0.438 (*p* = 0.001)
Virachip N IgG	0.583 (*p* < 0.001)	0.288 (*p* = 0.037)	0.068 (*p* = 0.637)

**Table A2:** Spearman correlation coefficients between all individual assays and age.

Assay	All Samples	PCR Positive	PCR Negative
Architect IgG	0.217 (*p* = 0.027)	0.033 (*p* = 0.819)	−0.131 (*p* = 0.350)
Liaison Trimetrics IgG	0.137 (*p* = 0.166)	0.010 (*p* = 0.943)	−0.305 (*p* = 0.026)
Cobas N-Protein	0.276 (*p* = 0.005)	−0.046 (*p* = 0.750)	0.125 (*p* = 0.373)
Cobas S-Protein	−0.127 (*p* = 0.239)	0.112 (*p* = 0.474)	−0.402 (*p* = 0.006)
cPass	0.062 (*p* = 0.531)	−0.141 (*p* = 0.323)	−0.321 (*p* = 0.019)
Lineblot SARS-CoV-2 NP	0.165 (*p* = 0.093)	0.073 (*p* = 0.612)	
Lineblot SARS-CoV-2 RBD	0.046 (*p* = 0.641)	0.007 (*p* = 0.962)	−0.252 (*p* = 0.069)
Lineblot SARS-CoV-2 S1	0.073 (*p* = 0.463)	0.018 (*p* = 0.899)	−0.170 (*p* = 0.225)
Virachip S1 IgG	0.126 (*p* = 0.207)	0.245 (*p* = 0.084)	−0.176 (*p* = 0.216)
Virachip RBD IgG	0.054 (*p* = 0.593)	0.230 (*p* = 0.104)	−0.149 (*p* = 0.298)
Virachip S2 IgG	0.191 (*p* = 0.054)	0.155 (*p* = 0.277)	−0.136 (*p* = 0.343)
Virachip N IgG	0.321 (*p* = 0.001)	0.182 (*p* = 0.201)	0.102 (*p* = 0.478)

**Table A3:** Spearman correlation coefficients between all individual assays and Ct values (PCR-positive only).

Assay	PCR Positive
Architect IgG	0.384 (*p* = 0.005)
Liaison Trimetrics IgG	0.078 (*p* = 0.584)
Cobas N-Protein	0.417 (*p* = 0.002)
Cobas S-Protein	0.143 (*p* = 0.360)
cPass	0.171 (*p* = 0.229)
Lineblot SARS-CoV-2 NP	0.370 (*p* = 0.007)
Lineblot SARS-CoV-2 RBD	0.059 (*p* = 0.682)
Lineblot SARS-CoV-2 S1	0.103 (*p* = 0.474)
Virachip S1 IgG	0.103 (*p* = 0.472)
Virachip RBD IgG	0.108 (*p* = 0.452)
Virachip S2 IgG	0.306 (*p* = 0.029)
Virachip N IgG	0.229 (*p* = 0.106)
